# Are Dimensions of Gender Inequality Uniformly Associated With Human Values?

**DOI:** 10.5964/ejop.2261

**Published:** 2021-05-31

**Authors:** Serena Stefani, Gabriele Prati

**Affiliations:** a Department of Psychology and Cognitive Science, University of Trento, Rovereto (TN), Italy; b Department of Psychology, University of Bologna, Cesena (FC), Italy; The Maria Grzegorzewska University, Warsaw, Poland

**Keywords:** gender, equality, values, dimensions, cross-sectional, women

## Abstract

A previous work of Schwartz and Rubel-Lifschitz (2009, https://doi.org/10.1037/a0015546) highlighted the association between human values and gender equality. However, gender equality is not a monolith. Indeed, it is a multidimensional phenomenon. We started from this multidimensionality to understand how the relative importance of human values varies through the different dimensions of Gender Equality Index (GEI)—namely work, money, knowledge, time, power, and health. We have designed a cross-national study based on secondary data analysis from international databases (i.e., European Social Survey [ESS] and GEI). Through the Bayesian correlational analysis of 18 European countries, findings revealed that 1) universalism, benevolence and self-direction are strongly and positively correlated to gender equality; 2) security, power and achievement are strongly and negatively correlated to equality while 3) conformity, tradition, stimulation, and hedonism have weak/non-significant correlation coefficients with gender equality. Relevance to cultural values and ideologies that support social equality are discussed. Furthermore, we find that some values are related to certain specific gender equality dimensions. Our results provide a more fine-grained analysis compared to previous findings, by outlining a more complex scenario.

There are different socio-cultural mechanisms related to gender roles (Eagly, Wood, & Diekman, 2000) and stereotypes (Fisk & Ridgeway, 2018) that legitimate unequal outcomes in a multitude of areas of gender experience. Therefore, it is essential to take gender equality's complexity into account in analyzing the beliefs, values, and attitudes linked with it. In the present study, we focus on the relationship between dimensions of gender inequality and Schwartz’s (1992) human values. Before discussing these associations in detail, we first present an overview of the dimensions of gender inequality and its implications. Then, we critically discuss the findings of Schwartz and Rubel-Lifschitz (2009) in light of a multidimensional framework on gender equality.

## Gender (In)Equality: A Multidimensional Phenomenon

Gender (in)equality is a complex and multidimensional phenomenon (Walby, 2004). Gender equality has multiple dimensions because inequalities arise from numerous interactions and feedback between actions at individual, family, community, and societal scales (Acker, 1990; Verloo, 2006). An action to promote an aspect of gender equality can undermine (and implicitly devalue) other areas of equality (Acker, 1990, 2000, 2006, 2009, 2012). A common example is the management of interplay between job and domestic work: the women's increasing participation in the labor market does not constitute actual work equality without redistribution of domestic work and equal gender pay (Kan, Sullivan, & Gershuny, 2011; Lewis, 1992; McGinnity & Whelan, 2009). So, there are many dimensions of gender equality and care must be taken into account to ensure an organic understanding of it. A fragmented overview can have repercussions on other sectors where inequality exists (O'Brien et al., 2019). For this purpose, the European Institute for Gender Equality (EIGE, 2017) identified and indexed six dimensions in which gender inequality manifests itself: in work, in the holding of money and economic position, in knowledge and education, in the use of free time, in socio-political power and in health.

In addition to the multitude of aspects of life in which gender inequality reverberates, it is interesting to note how the harmful effects are not only manifested at microlevel, but also at a social and economic macrolevel. It has been in fact studied that gender inequality reflects impoverished society at several junctures. Countries with greater gender inequality have slower economic growth and more pronounced economic inequalities (Cuberes & Teignier, 2016; Mitra, Bang, & Biswas, 2015; Seguino, 2000), lower stocks of democracy (Beer, 2009; Inglehart, Norris, & Welzel, 2002) larger corruption in political institution (Dollar, Fisman, & Gatti, 2001; Swamy, Knack, Lee, & Azfar, 2001) and lesser educational attainment (Hirshfield & Glass, 2018; Knowles, Lorgelly, & Owen, 2002). Instead, greater gender equality at country level is associated with ecological footprints (Dietz, Kalof, & Stern, 2002; McKinney & Fulkerson, 2015), job satisfaction (Mencarini & Sironi, 2010) and general well-being (Bjørnskov, Dreher, & Fischer, 2008; Fischer, Bjørnskov, & Dreher, 2007). These characteristics are known to be linked to the system of human values identified by Schwartz (1992, 2006, 2007b), that is, they influence the importance that individuals attach to each value (Arciniega & González, 2005; Arikan & Bloom, 2015; Dietz et al., 2002; Inglehart, Basanez, & Moreno, 1998; Öcal, Kyburiene, & Yiğittir, 2012; Yeganeh & May, 2011). Gender inequality has also been associated with the human value system (Schwartz & Rubel-Lifschitz, 2009). An overview of Schwartz human values system, along with Table S1 and Figure S1, is provided in Supplementary Materials: SM1.

## Previous Results on Association Between Human Values and Gender Equality

Gender equality is associated with relative importance that people attribute to basic human values (Schwartz & Rubel-Lifschitz, 2009). Using archival data (Study 1) and a sample of students (Study 2) Schwartz and Rubel-Lifschitz (2009) tested the effects of gender equality on the overall importance of the 10 values for women and men (Hypotheses 1 and 2) and effects of gender equality on the size of sex differences in the importance of values (Hypothesis 3–6). According to both Study 1 and Study 2, greater gender equality within countries was associated with the importance of values of benevolence, universalism, self-direction, stimulation and hedonism (anxiety-free values), while in countries with less gender equality greater importance was given to power, achievement, security, conformity and tradition values (anxiety-based values). It was also discovered that the preference for some values had a divergent effect for the two sexes based on the gender inequality of the country: Women gave more importance to values of benevolence and universalism more than men did, whereas men valued power, achievement, and stimulation more than women. Moreover, where gender equality was higher, sex differences in these values increased. Sex differences in tradition, conformity, security, hedonism, and self-direction values were not be affected by increases in gender equality.

In their work, Schwartz and Rubel-Lifschitz (2009) highlighted the association between two clusters of values and gender inequality; however, a unique gender inequality index was used in their study, neglecting the multidimensionality of the phenomenon. Since gender equality is a multidimensional construct, it is not clear whether these relationships would hold when considering multiple dimensions of gender equality.

## Hypothesis

Our study is based on the results of Schwartz and Rubel-Lifschitz’s (2009) work, and aspires to deepen the link between values and gender inequality in the various areas in which gender inequality exists. Our aim is to understand which values deviate from the pattern identified by Schwartz and Rubel-Lifschitz (2009) when it comes to gender inequality in defined contexts, such as in the workplace, monetary, health, in places of institutional political power, in leisure time and in education. To accomplish this, we have designed a cross-national study based on secondary data analysis from international databases: European Social Survey (ESS) and Gender Equality Index (GEI). The exploratory focus of this study fills the gap in the literature on the relationship between multiple dimensions of gender inequality and human values. Based on the study of Schwartz and Rubel-Lifschitz (2009), we address the following research questions:

RQ1: Does the importance of power, achievement, security, conformity, and tradition values decrease with greater scores on dimensions (i.e., work, money, knowledge, time, power, and health) of gender equality?

RQ2: Does the importance of benevolence, universalism, stimulation, hedonism, and self-direction values increase with greater scores on dimensions (i.e., work, money, knowledge, time, power, and health) of gender equality?

## Method

### Data

Data were obtained creating ad hoc datasets through data linkage between two international databases: Round 8 of the ESS and GEI of the European Institute for Gender Equality (EIGE). Round 8 of the ESS includes representative samples of the population aged 15 and older (44,387 participants, 47.4% men and 52.6% women). We used data from 18 total countries^1^1ESS collects data for 23 European countries, while GEI holds data for 28 countries. Eighteen total countries were matching in both datasets.; The countries included were: Austria, Belgium, Czech Republic, Germany, Estonia, Finland, France, Hungary, Ireland, Italy, Lithuania, Netherlands, Poland, Portugal, Slovenia, Spain, Sweden, and the United Kingdom. Data from ESS were collected using face-to-face interviews held between 2016 and 2017.

The EIGE is a European Institute that supports the development and implementation of evidence-based gender equality policies and legislation and shows the different outcomes of those policies for women and men. It provided data at aggregate level on gender gaps between women and men within 28-EU countries. We used only the same 18 countries of ESS. We adopted data released in 2017 and collected between 2014 and 2016.

### Measures

In this study, two measures were used: human values and gender equality. Human values were measured by ESS thorough Portrait Values Questionnaire (PVQ; Schwartz et al., 2001), shortened and revised in a 21-items scale. The PVQ includes short verbal portraits of different people, gender matched with the respondent. Each portrait describes a person’s goals, wishes or aspirations that point implicitly to the importance of a single value type. For each portrait, the participants answer the question “How much like you is this person?” by choosing one of the Likert scale boxes from 1 to 6 where 1 is “*not like me at all*” and 6 “*very much like me*.” For example: “Being very successful is important to her. She likes to impress other people.” Thus, respondents’ own values are inferred from their self-reported similarity to people who are described in terms of particular values. Such an instrument is more adaptable than the Schwartz Values Survey (SVS) for this kind of survey because it is shorted (SVS has 56 items) and more suitable for use with all segments of the population, including those with little or no formal education. PVQ permits use of cross-culturally validated theory to predict and explain variation in macro phenomena like inequalities (Schwartz et al., 2001). The internal reliability of the values in the 21 items PVQ ranged from .39 for tradition to .79 for hedonism. Each value is based on two items only (only universalism is based on three items). The associations of these value scores with variables as political orientations, attitudes, interpersonal trust, and social involvement support their validity (Schwartz, 2007a, 2010). We centered each person’s responses on their own mean values to eliminate individual differences in use of the response scale. Such corrections convert absolute value scores into scores that indicate the relative importance of each value in the value system—that is, the individual’s value priorities (Schwartz, 1992). Each mean was then aggregated at country level.

For gender equality, we used the GEI provided by the EIGE. GEI is a complex gender equality indicator to assess the status and monitor the progress of this phenomenon across the EU over time (EIGE, 2017). The GEI has an overall score and six main domains (work, money, knowledge, time, power, and health). The GEI assigns scores for Member States between 1 for total inequality and 100 for full equality.

### Statistical Analysis

We tested the relationships between human values and dimensions of gender equality by performing Bayesian correlations (i.e., Bayesian inference about Pearson correlation coefficient). Since for some values—namely tradition, power, and self-direction—the alpha level was very low (< .50), we made separate analyzes for each of the items. We computed the posterior median of the correlation coefficient and the corresponding Bayesian 95% credible intervals (CIs). This interval is interpreted as having a 95% probability of including the true correlation. When the 95% credible interval does not contain zero, this indicated a statistically significant correlation. Compared with the standard frequentist test, the Bayesian approach has several practical advantages, especially when the sample size is small or when parameters are not normally distributed (e.g., Lee & Song, 2004; McNeish, 2016; Stegmueller, 2013). Another advantage of the Bayesian approach is the use of CIs rather than on hypothesis testing by *p* values. The analyses were conducted using IBM SPSS Statistics (Version 25). The magnitude (i.e., effect size) of the correlation coefficients was evaluated using the guidelines provided by Cohen (1988): about .10 = small effect, about .30 = medium, about .50 = large.

## Results

**Figure 1:**
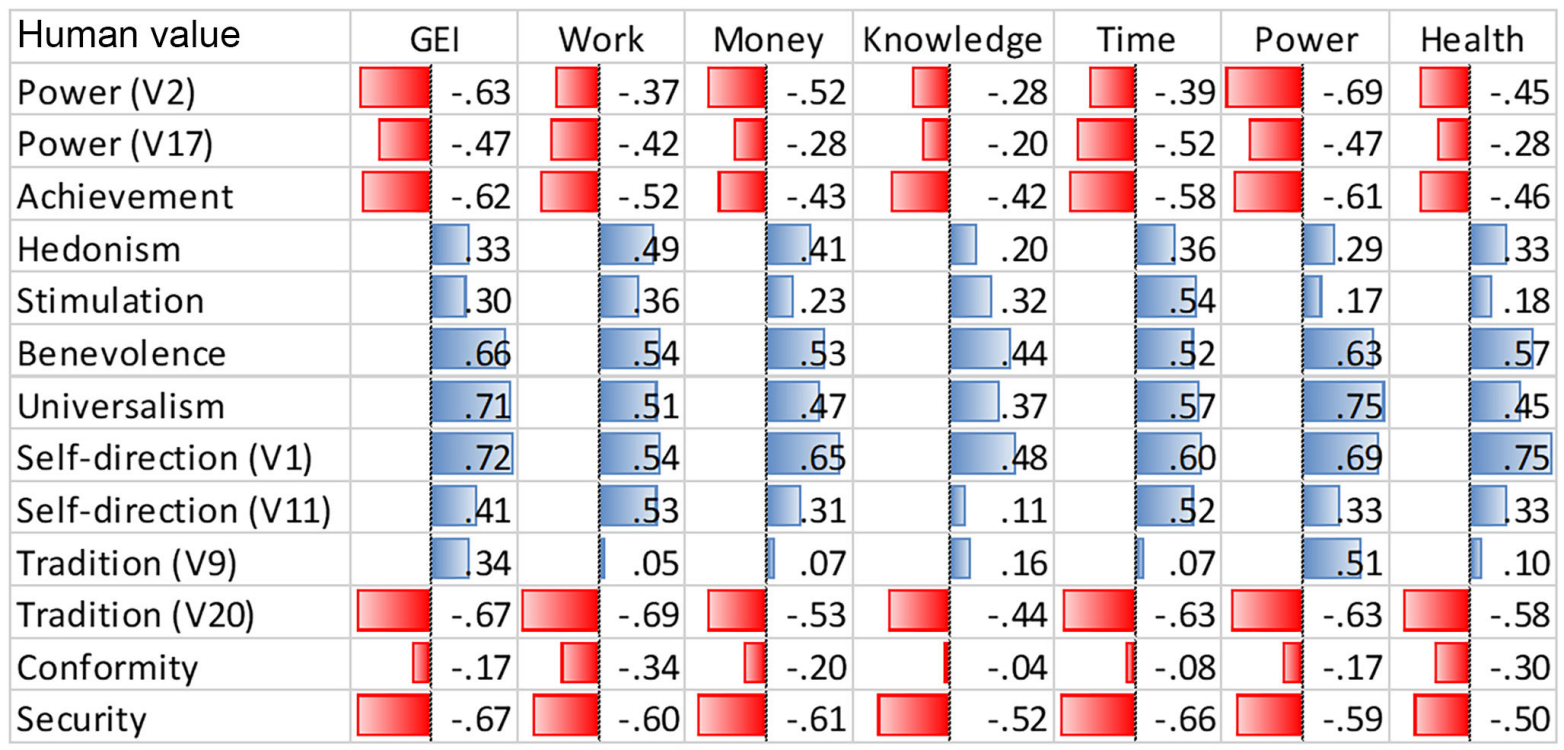
Representation of Correlation Coefficients Using Data Bars *Note*. GEI = Gender Equality Index; V = Variable (item of the scale and its number).

Combining the data from the two databases, we were able to obtain complete information for 18 European countries. Figure 1 and Table 1 report results of the analyses across the 18 countries. In columns there are values and relative items (centered mean aggregated by country), while in the rows the overall gender inequality and each dimension of it are listed. Figure 1 displays correlation coefficients using data bars: A longer bar represents a higher correlation coefficient, while blue and red bars represent positive and negative correlation coefficients, respectively.

**Table 1:** Posterior Distribution Characterization (and 95% Credibility Intervals) for Pairwise Correlations Among Human Values and Gender Equality Domains

Human value	GEI		Work		Money		Knowledge		Time		Power		Health	
	*r*	95 % CI	*r*	95 % CI	*r*	95 % CI	*r*	95 % CI	*r*	95 % CI	*r*	95 % CI	*r*	95 % CI
Power (V2)	**−.63**	[−.25, −.84]	−.37	[.07, −.69]	**−.52**	[−.11, −.78]	−.28	[.17, −.63]	−.39	[.05, −.70]	**−.69**	[−.35, −.87]	**−.45**	[−.02, −.74]
Power (V17)	**−.47**	[−.04, −.75]	−.42	[.02, −.72]	−.28	[.18, −.63]	−.20	[.25, −.57]	**−.52**	[−.10, −.78]	**−.47**	[−.04, −.75]	−.28	[.17, −.63]
Achievement	**−.62**	[−.24, −.83]	**−.52**	[−.10, −.78]	−.43	[.01, −.73]	−.42	[.02, −.72]	**−.58**	[−.18, −.81]	**−.61**	[−.22, −.83]	**−.46**	[−.03, −.74]
Hedonism	.33	[.67, −.11]	**.49**	[.76, .06]	.41	[.71, −.03]	.20	[.58, −.24]	.36	[.68, −.08]	.29	[.63, −.16]	.33	[.67, −.11]
Stimulation	.30	[.64, −.15]	.36	[.68, −.09]	.23	[.59, −.22]	.32	[.65, −.13]	**.54**	[.79, .13]	.17	[.56, −.27]	.18	[.56, −.27]
Benevolence	**.66**	[.85, .29]	**.54**	[.79, .13]	**.53**	[.79, .12]	**.44**	[.73, .01]	**.52**	[.78, .10]	**.63**	[.84, .25]	**.57**	[.81, .17]
Universalism	**.71**	[.88, .37]	**.51**	[.77, .09]	**.47**	[.75, .04]	.37	[.68, −.08]	**.57**	[.81, .17]	**.75**	[.90, .45]	**.45**	[.74, .02]
Self−direction (V1)	**.72**	[.89, .39]	**.54**	[.79, .13]	**.65**	[.85, .28]	**.48**	[.76, .06]	**.60**	[.82, .21]	**.69**	[.87, .34]	**.75**	[.90, .45]
Self−direction (V11)	.41	[.71, −.03]	**.53**	[.79, .12]	.31	[.65, −.13]	.11	[.51, −.33]	**.52**	[.78, .10]	.33	[.66, −.12]	.33	[.66, −.12]
Tradition (V9)	.34	[.67, −.11]	.05	[.46, −.38]	.07	[.47, −.37]	.16	[.54, −.29]	.07	[.48, −.36]	**.51**	[.77, .08]	.10	[.50, −.34]
Tradition (V20)	**−.67**	[−.31, −.86]	**−.69**	[−.35, −.87]	**−.53**	[−.12, −.79]	**−.44**	[−.00, −.73]	**−.63**	[−.25, −.84]	**−.63**	[−.25, −.84]	**−.58**	[−.19, −.82]
Conformity	−.17	[.28, −.55]	−.34	[.11, −.67]	−.20	[.25, −.57]	−.04	[.39, −.46]	−.08	[.36, −.48]	−.17	[.27, −.55]	−.30	[.16, −.64]
Security	**−.67**	[−.31, −.86]	**−.60**	[−.21, −.83]	**−.61**	[−.23, −.83]	**−.52**	[−.10, −.78]	**−.66**	[−.30, −.86]	**−.59**	[−.19, −.82]	**−.50**	[−.08, −.77]

*Note*. *N* = 18 European countries. The 95 % credible interval of coefficients in boldface did not include zero. GEI = Gender Equality Index; V = Variable (item of the scale and its number).

As regards to the directionality of correlations (Table 1 and Figure 1), we observe that all the coefficients are negative for security, conformity, tradition, achievement, and power values, while they are positive for benevolence, universalism, self-direction, stimulation and hedonism values. The values, however, show different degrees of statistical significance which reflect peculiarities relating to each dimension of gender inequality.

Looking at the values associated negatively with gender equality, negative and statistically significant correlations were found between GEI and security, *r =* −.67, 95% CIs [−.31, −.86], achievement, *r =* −.62, 95% CIs [−.24, −.83], both items of power, V2 *r =* −.63, 95% CIs [−.25, −.84] and V17 *r =* −.47, 95% CIs [−.04, −.75], and the second item (V20) of tradition (“Important to follow traditions and customs”)*, r =* −.67, 95% CIs [−.31, −.86]. In this case, security value reported the strongest negative coefficient and was significantly associated with all dimensions of gender equality. GEI was positively correlated with benevolence, *r =* .66, 95% CIs [.85, .29], universalism, *r =* .71, 95% CIs [.88, .37], and the first item (V1) of self-direction, *r =* .72, 95% CIs [.89, .39]. Benevolence and the first item (V1) of self-direction (“Important to think new ideas and being creative”) were significantly and positively associated with all the dimensions of gender equality. Also, universalism was significantly associated with all the dimensions of gender equality except for gender inequality in knowledge. Gender inequality in knowledge was significantly associated with security, *r =* −.52, 95% CIs [−.10, −.78], the second item (V20) of tradition, *r =* −.44, 95% CIs [−.00, −.73], benevolence, *r =* .44, 95% CIs [.73, .01], and first item (V1) of self-direction, *r =* .48, 95% CIs [.76, .06].

As far as power and achievement values are concerned, significant negative correlations with GEI and power dimensions are shown, while their associations with gender inequality in knowledge were not significant. However, some peculiarities were present. Only achievement correlated with work dimension, *r =* −.52, 95% CIs [−.10, −.78], and only the first item (V2) of power correlated with gender equality in money, *r =* −.52, 95% CIs [−.11, −.78]. In addition, gender equality in health was significantly correlated with the first item (V1) of power (“Important to be rich, have money and expensive things”), *r =* −.45; 95% CIs [−.02, −.74], but not with the second item (V17) of power (“Important to get respect from others”), *r =* −.28, 95% CIs [.17, −.63]. Instead, the second item (V17) of power was negatively associated with gender equality in time, *r =* −.52, 95% CIs [−.10, −.78]. Stimulation correlated positively with gender equality in time, *r =* .54, 95% CIs [.79, .13], while hedonism was significantly related to gender equality in work, *r =* .49, 95% CIs [.76, .06]. The first item (V9) of tradition was significantly associated with gender equality in power, *r =* .51, 95% CIs [.77, .08], while the second item (V20) was negatively related to all the dimensions of gender equality. Conformity was not significantly associated with any dimension of gender equality.

## Discussion

The aim of the current work was to investigate the relationships between multiple dimensions of gender inequality and human values. It was found that the endorsement of human values is linked to the degree of gender equality in a country. According Schwartz and Rubel-Lifschitz (2009) outcomes, values of universalism, benevolence, self-direction, stimulation, and hedonism are considered more important by countries where there is greater gender equality, while power, achievement, security, conformity and tradition are the values that assume greater importance in more gender unequal countries. This pattern refers to the relationship of values with anxiety: anxiety-free values and anxiety-based values. However, it is important to emphasize that gender inequality is a multidimensional phenomenon and appears in different forms, from the scarce presence of women in institutions to the pay gap, up to the uneven amount of free time between genders. Therefore, a possibility is that the values elicited in a society vary according to the dimension of gender inequality referred to. Our results provided support for this perspective. Overall, our fine-grained analysis seems to identify a different clustering, although with some reservations. An overview of the results is given in Table S2, Supplementary Materials: SM2. The relationship between values and gender inequality changes according to the dimension considered and it is based on the significance/magnitude of the correlations. Based on our results, 1) universalism, benevolence, and self-direction were strongly and positively correlated to gender equality; 2) security, power, and achievement were strongly and negatively correlated to equality and; 3) conformity, stimulation, tradition, and hedonism had inconsistent/weak/non-significant correlations coefficients with gender equality. Our analysis also revealed that the value index changes based on the items content (e.g., tradition value), hence, it is important to take the formulation of items into account (explained in detail below). The results of our study indicated that in the structure of relations among motivationally distinct values, the key axis involved in gender equality is the one going from self-direction to universalism up to benevolence (two values of self-transcendence and one of openness) and the one going from achievement to power up to security value (two values of self-enhancement and one of conservation). So, gender equality involves both values of self-transcendence and openness to change, while gender inequality includes both values of self-enhancement and conservation. Cultural values of conservatism and autonomy had already been associated with the gender gap within countries, the former in a positive sense and the latter negatively (Yeganeh & May, 2011). The conservatism value is characterized by social order, respect for tradition, propriety, and family security. The security value, which is associated with more unequal countries in the present research, fits well with characteristics of conservatism, and it is no coincidence that in conservative cultures the beliefs that men are better political leaders and have more right to a job than women are widespread (Inglehart et al., 2002). By contrast, self-direction is conceptually very close to the cultural value of autonomy which was associated with greater gender equality in the study of Yeganeh and May (2011). Autonomy (which emphasizes the opportunity to pursue one's own ideas and intellectual directions) is the prevailing cultural value of more gender-equal societies. However, it is important to differentiate that in our study the first item (V1) of self-direction (“thinking of new ideas and being creative”) was significantly related to all the dimensions of gender equality, while the second item (V11) of self-direction (“importance of making own decisions and being free”) was linked to gender equality in work and time only. The item V1 of self-direction refers to the self-expression through innovation and denotes a certain mental openness and progressist thinking which is typical of societies that tend to embrace a broader sense of inclusion on issues of abortion/divorce/homosexuality (Inglehart & Baker, 2000; Inglehart et al., 2002; Welzel & Inglehart, 2005). Differently, the second item of self-direction seemed to be more tied to an emancipatory measure. Above all, the importance of freedom may be directly reconnected to work and leisure gender balance.

Although there are no previous studies investigating gender equality in relation to the values of self-transcendence, such values fit very well with political issues related to equality. Gender equality is a phenomenon inscribed in the broader concept of social equality, a concept whereby all individuals must have the same state of social respectability by breaking down issues of class, gender, race, and socioeconomic status. The aims and values of equality, solidarity and social justice refer to values of universalism and benevolence historically supported by liberal leftist ideologies (Jost, Nosek, & Gosling, 2008; Lipset, 1960; Lipset & Rokkan, 1967). In fact, center-left supporters have given priority to universalism and values of benevolence while center-right voters have given higher priority to the values of power, achievement, security, and conformity in an Italian sample (Caprara, Schwartz, Capanna, Vecchione, & Barbaranelli, 2006; Caprara, Schwartz, & Vecchione, 2010); similar findings were found in another cross-country study that included 20 countries (Piurko, Schwartz, & Davidov, 2011).

The values of conformity, hedonism, tradition, and stimulation make up the group of values that were weakly, inconsistently, or non-significantly associated with the GEI. It is interesting to note that tradition and conformity “share an underlying motivation to avoid threats and anxiety” (Schwartz & Rubel-Lifschitz, 2009, p. 174), whereas hedonism, and stimulation express anxiety-free motivations (Schwartz, 2006). However, the role of tradition is quite controversial because one item correlates positively and the other negatively with gender equality and related dimensions. At first, we had averaged the item scores, but the internal consistency was low so we decided to uncouple them. The analyses conducted on the two separated items of tradition revealed that the second item of tradition (V20: “Important to follow traditions and customs”) was significantly and negatively associated with all gender equality dimensions, while the first item (V9: “Important to be humble and modest, not draw attention”) was not associated with any of the dimensions of gender equality, except for a positive correlation with power. The content of item V20 of tradition seems more associated with the idea of a traditionalist society that defends the status quo, so more averse to gender equality at all. Differently, the positive association between item V9 of tradition and dimension of power could highlight how in societies where more women occupy managerial/political positions, the leadership style assumes less stereotypical masculine-agentic characteristics (such as self-promotion or dominance) in favor of humility and modesty (Eagly & Karau, 2002; Rudman, 1998). Indeed, descriptions of leadership roles are highly imbued with cultural masculinity (Atwater, Brett, Waldman, DiMare, & Hayden, 2004) in which the role of leader is almost incompatible with the female gender (Eagly & Karau, 2002; Schein, 2001). Schein (2001) labeled this phenomenon as the “think manager—think male” effect. Nonetheless, with greater gender equality in power roles, these biases could fade. These results suggest that the formulation of the items could change the relationship even if both items are theoretically embedded in the same value.

Hedonism value was related to gender equality in work, while the value of stimulation was associated with greater gender equality in time. Conformity was not associated with any dimension of gender equality. The value of hedonism is specifically associated with fairer gender working societies, so the equal inclusion of women in work can increase the national average importance that the population attaches to direct gratification and in the pursuit of pleasure. Indeed, women have always been disadvantaged in the labor market and still they are (Arulampalam, Booth, & Bryan, 2007; Betti, 2018; EIGE, 2017; Sundötrom, 1991). Without deploying strong commitment and efforts, females—as a disadvantaged social group—have less expectations of success in the workplace and they are much more concerned about demonstrating their abilities (Marini, Fan, Finley, & Beutel, 1996). The data found that workers in higher social class positions attach more importance to the intrinsic aspects of work, (opportunity for self-expression, interest-value of work) while those in lower social class positions attach more importance to extrinsic rewards, such as high wages (Centers & Bugental, 1966). Greater gender equality in work would allow women greater tranquility and fewer worries about the income, which could explain the rise of hedonism in this dimension.

Another specific association is between stimulation (e.g., novelties and challenges, need for variety and stimuli) and gender equality in leisure time and related activities. The dimension of time considers both the large disparity in the division of care work for children / elderly and the time to devote to cultural activities, sports, and volunteering. The disparity in the division of care and assistance work could negatively affect the happiness of women (Mencarini & Sironi, 2010) limiting their free time to devote to more enjoyable and stimulating activities (e.g., playing sports). Indeed, physical activity and sport had already been shown to be linked to the importance of stimulation (Souchon, Bardin, Perrissol, & Maio, 2015).

The three clusters of values identified by this study reflect a bidirectional shaping (citizens-institutions) of needs and beliefs based on the national gender gap. Countries for which values of universalism, benevolence, self-direction are important are negatively affected when these values are threatened and are happier when they can enjoy their full social application, in terms of egalitarian policies and non-discriminatory actions. The construction of the judgment on the facts and on the consequences that derive from them is based on the relative importance that people attribute to values, which perform the function of reference criteria. Although people are often unaware of the impact of values on daily decisions, the evaluation of actions to reduce or maintain gender inequality is filtered by the groups of values that we have identified here. The motivational continuum of values of self-direction, universalism, and benevolence positively directs actions aimed at promoting gender equity and transcending one's selfish interests to struggle social injustices, as well as trusting one's own judgment and feeling at ease in the face of the diversity of existence. Otherwise, the motivational continuum of values of achievement, power, and security, steer positive attitudes to social superiority and esteem, contributing to the solidity of the hierarchy based on patriarchy. In particular, security and power—through the control of gender relations and the male monopoly of resources—are the values mostly in charge of preventing social changes. However, it is important to underline that, also in this case, the meaning of the items affects the correlations. For example, Item V2 of power (“Important to be rich, have money and expensive things”) is significantly related to the money dimension of gender equality, while the second item (V17: “Important to get respect from others”) is not. The relationship could be explained by the direct verbal reference to the money in the first item (V2) which is missing in the second one.

Although this work has proved to be important and innovative, it has some limitations that must be acknowledged. Statistical power of this study is limited by the low number of countries involved (*n* = 18). It is possible that, with a higher number of countries, we would have found other significant small correlations between human values and gender equality. Despite this, we would exclude substantial changes in the results, since 18 countries are enough to establish a realistic trend using Bayesian correlations (e.g., Lee & Song, 2004; McNeish, 2016; Stegmueller, 2013). Further studies are also needed to establish the generalization of these results beyond the European Union. The internal consistency of some items was low hence we have conducted a separated analysis for each item. This type of procedure has highlighted the limit of values measures: relationships changed according to the distinctive formulation of items. Therefore, we are not able to establish if some values are wholly tied to country-level gender equality. It depends on the aspect investigated by the items. However, by recognizing such items formulation bias it is possible to overcome this limit and make accurate interpretations.

Most of the studies that have investigated the relationship between values and gender equality have focused on cross-sectional analysis (Schwartz & Rubel-Lifschitz, 2009; Yeganeh & May, 2011) and our study is part of this chain, as this type of investigation is suitable for investigating the relationships between indagated variables, generating new knowledge at a theoretical level. Other works will confirm these findings through multiple-panel experiments. Future research may incorporate additional variables (economic development and inequality, unemployment rate, social and institutional trust) to better understand which processes and mechanisms mediate the relations between values and gender inequality.

### Conclusion

This research has contributed in an essential way to broaden the debate on gender inequality and human values, highlighting how gender inequality is not a uniform phenomenon, but stands out on different configurations. These configurations proved to be closely related to Schwartz’s human values system. This research provides a fine-grained analysis of the relationships among human values and gender inequality dimensions.

## Notes

For this article the following Supplementary Materials are available via the PsychArchives repository (for access see Index of Supplementary Materials below):

- SM1: Overview of Schwartz human values system, Table S1 and Figure S1.
- SM2: Overview of the results is given in Table S2.
